# Molecular Checkpoints of Nucleic Acid Sensing in Tumor Cells: Linking Innate Immune Evasion to Precision Cancer Immunotherapy

**DOI:** 10.3390/ijms27219417

**Published:** 2026-10-22

**Authors:** Yan Wang, Bowei Cao, Huili Lu, Zhicheng Wang, Yanli Qin

**Affiliations:** 1 Department of Geriatrics, Huashan Hospital, Fudan University, Shanghai 200040, China; 09211220054@fudan.edu.cn; 2 Department of Transfusion Medicine, Huashan Hospital, Fudan University, Shanghai 200040, China; 17786358546@163.com; 3 School of Pharmacy, Shanghai Jiao Tong University, Shanghai 200240, China; roadeer@sjtu.edu.cn; 4 Department of Infectious Diseases, Shanghai Key Laboratory of Infectious Diseases and Biosafety Emergency Response, National Medical Center for Infectious Diseases, Huashan Hospital, Shanghai Medical College, Fudan University, Shanghai 200040, China

**Keywords:** tumor intrinsic innate immunity, nucleic acid sensing, cGAS–STING, RIG-I/MDA5, TREX1, ADAR1, ENPP1, immune checkpoint blockade, precision immune oncology

## Abstract

Tumor cells possess nucleic-acid sensing systems that detect aberrant DNA and RNA arising from genomic and transcriptomic dysregulation. Cytosolic DNA and RNA sensors, together with endosomal nucleic acid sensors, can induce type I interferons, inflammatory chemokines, antigen presentation programs, and immune cell recruitment. Yet many tumors remain immunologically silent despite harboring potentially immunostimulatory nucleic acids, indicating that malignant cells can restrain these pathways at multiple levels. Nucleic acid clearance, selective RNA editing and remodeling, extracellular second messenger degradation, and epigenetic silencing can suppress these responses. These regulatory layers may favor immune-poor tumor states and limit responsiveness to immune checkpoint blockade. Therapeutic reactivation must be spatially and temporally controlled, because persistent signaling can undergo maladaptive rewiring. We review these molecular brakes, therapeutic strategies to restore innate sensing, and a proposed biomarker framework integrating sensor competence, suppressor activity, and functional immune output. We distinguish the effects arising within tumor cells from those mediated by sensing mechanisms in neighboring host cells and discuss cellular and spatial context, signaling dynamics, and safety. The proposed framework for matching treatments to patients remains investigational and requires prospective clinical validation.

## 1. Introduction

Cancer immunotherapy has transformed the treatment landscape across multiple malignancies, with immune checkpoint blockade (ICB) targeting programmed cell death protein 1 (PD-1), programmed death-ligand 1 (PD-L1), and cytotoxic T-lymphocyte-associated protein 4 (CTLA-4) representing one of its most successful clinical advances [1]. Nevertheless, durable clinical benefit remains confined to a subset of patients, and both primary and acquired resistance to immune checkpoint blockade are common [2]. The baseline immune contexture of the tumor microenvironment is an important determinant of therapeutic response [3]. Tumors characterized by active antigen presentation, interferon-responsive programs, chemokine expression, and substantial intratumoral T cell infiltration are generally more likely to respond to PD-1/PD-L1 blockade, whereas tumors with sparse or excluded T cell infiltration are more frequently associated with resistance [4,5]. Understanding the upstream mechanisms that determine whether a tumor becomes immunologically visible is therefore central to improving cancer immunotherapy.

The terms immunologically “cold” and “hot” describe broad immune phenotypes rather than fixed tumor categories. Here, cold includes poorly infiltrated or immune-excluded tumors, whereas hot denotes an inflamed, T cell infiltrated state; neither designation alone establishes functional nucleic acid sensing or predicts response in an individual patient [4,5].

Innate nucleic acid sensing represents one such upstream regulatory layer. Pattern recognition receptors that detect aberrant or mislocalized deoxyribonucleic acid (DNA) and ribonucleic acid (RNA) were initially characterized mainly in the context of host defense against infection and antiviral immunity [6]. It is now increasingly clear, however, that malignant cells can retain functional pathways for intracellular nucleic acid sensing [7,8]. Cancer cells commonly exhibit genomic instability, chromosome missegregation, mitochondrial dysfunction, aberrant transcription and RNA processing, and derepression of repetitive elements. These abnormalities can generate or expose endogenous DNA and RNA species capable of engaging innate immune sensors [7,9,10]. Cytosolic DNA is sensed primarily through cyclic GMP–AMP synthase (cGAS), a DNA sensor, and its downstream signaling adaptor stimulator of interferon genes (STING) [11], whereas cytoplasmic immunostimulatory RNA can be detected by the retinoic acid-inducible gene I (RIG-I)-like receptors (RLRs), including RIG-I and melanoma differentiation-associated protein 5 (MDA5), which signal through the mitochondrial antiviral-signaling protein (MAVS) adaptor [9,12]. Endosomal Toll-like receptor (TLR) 3, TLR7/8, and TLR9 provide an additional compartmentalized system for sensing double-stranded RNA (dsRNA), single-stranded RNA (ssRNA) and RNA-derived ligands, and unmethylated cytosine-phosphate-guanine (CpG)-rich single-stranded DNA, respectively [13].

Activation of these pathways can have consequences that extend well beyond the tumor cell. Nucleic acid sensing within tumor cells can induce type I interferons and inflammatory chemokines, enhance antigen processing and presentation, and promote the recruitment and activation of dendritic cells, natural killer (NK) cells, and T cells [9,14,15,16]. In this way, intracellular abnormalities within malignant cells can be translated into signals that reshape the composition and functional state of the surrounding immune microenvironment [9,14,15,16]. Tumor cells should therefore be considered not only as targets of immune surveillance but also as active participants in shaping the initiation and maintenance of antitumor immune responses.

This raises an apparent paradox. Cancer cells frequently harbor genomic and transcriptomic abnormalities capable of generating endogenous ligands for innate immune sensors, yet many tumors remain poorly inflamed or immunogenic. How, then, do malignant cells avoid sustained activation of nucleic acid sensing within the tumor cell? Emerging evidence indicates that cancer cells restrain these pathways at multiple levels by degrading immunostimulatory nucleic acids, editing or processing endogenous RNA, limiting second-messenger propagation, and epigenetically silencing sensing components [17,18,19,20,21,22]. Representative molecular brakes include the nucleases three-prime repair exonuclease 1 (TREX1) and 5′–3′ exoribonuclease 1 (XRN1) [17,18]; RNA editing and processing factors such as adenosine deaminase acting on RNA 1 (ADAR1), DExH-box helicase 9 (DHX9), and DEAD-box helicase 6 (DDX6) [19,20,21]; ectonucleotide pyrophosphatase/phosphodiesterase 1 (ENPP1), which hydrolyzes extracellular cyclic GMP–AMP (cGAMP) [22]; and epigenetic repression of cGAS–STING and related sensing pathways [23].

These mechanisms can be viewed as molecular brakes on innate immune signaling in tumor cells. This framework also suggests a therapeutic opportunity: endogenous immune surveillance may be restored not only through exogenous agonism but also by releasing the molecular restraints that prevent cancer cells from sensing their own aberrant nucleic acids.

In this review, we examine nucleic acid sensing in tumor cells as an upstream regulator of cancer immunity. We first summarize the major DNA and RNA sensing pathways in malignant cells, including cytosolic and endosomal systems. We then discuss the mechanisms that suppress these pathways, including nucleic acid clearance, RNA editing and processing, degradation of intercellular second messengers, and epigenetic silencing. We discuss how these processes contribute to interferon suppression, defective antigen presentation, impaired immune-cell recruitment, and resistance to ICB. We then consider therapeutic approaches aimed at restoring innate immune activity in tumor cells, with particular emphasis on direct sensor activation, emerging forms of innate immune checkpoint blockade, and rational combinations with established immunotherapies. Finally, we discuss biomarker strategies and the challenges involved in defining immune states within tumors across cellular, spatial, and temporal contexts. Integrating sensor competence, suppressor activity, and functional immune output provides a framework for further investigation rather than an approach for selecting treatments that has been clinically validated.

## 2. Nucleic Acid Sensing in Tumor Cells and Cancer Immunity

Malignant cells are exposed to aberrant DNA and RNA generated by genomic instability, altered transcription, mitochondrial dysfunction, derepression of repetitive elements, and defective RNA processing. These nucleic acids can activate innate immune sensors within tumor cells, thereby linking intracellular abnormalities to type I interferon production, chemokine release, antigen processing and presentation, and remodeling of the local immune microenvironment.

Three sensing compartments are most relevant to this review: cytosolic DNA sensing through cGAS–STING, cytoplasmic RNA sensing through RIG-I-like receptors (RLRs), and nucleic acid sensing in endosomes mediated by TLR3, TLR7/8, and TLR9 [11,12,24]. Although these systems differ in ligand specificity, subcellular localization, and proximal signaling adaptors, they ultimately converge on overlapping transcriptional programs controlled by interferon regulatory factors 3 and 7 (IRF3 and IRF7) and nuclear factor kappa B (NF-κB), thereby promoting the production of type I interferons and other inflammatory mediators [6]. In cancer, the biological consequences of these pathways are highly context-dependent, varying with the cellular compartment, signaling strength, and duration [25].

### 2.1. DNA Sensing

The cGAS–STING pathway plays a major role in cytosolic DNA sensing in cancer cells. When double-stranded DNA (dsDNA) aberrantly accumulates in the cytosol, cGAS binds dsDNA and catalyzes the synthesis of the second messenger 2′3′-cGAMP [11,26]. cGAMP subsequently binds and activates STING, leading to TANK-binding kinase 1 (TBK1) recruitment and IRF3 activation and thereby inducing type I interferons, together with broader inflammatory signaling [27].

In tumors, cytosolic DNA can arise from chromosome missegregation and micronuclear rupture or mitochondrial DNA release [28,29,30]. Chromosomal instability is particularly relevant because chromosome segregation errors can generate micronuclei whose rupture exposes micronuclear chromatin to cytosolic cGAS [29,30]. In this way, tumor-cell genomic instability can be coupled to cell-intrinsic innate immune signaling [30].

Activation of cGAS–STING within tumor cells can remodel the tumor microenvironment by inducing type I interferons and chemokines such as C-C motif chemokine ligand 5 (CCL5) and C-X-C motif chemokine ligand 10 (CXCL10) that attract T cells, thereby promoting the accumulation and activation of effector immune cells, including T cells and natural killer (NK) cells [14,31]. STING signaling within tumor cells can also increase the expression of interferon-stimulated genes (ISGs) and components involved in antigen processing and presentation, thereby providing a mechanistic link between intracellular DNA abnormalities and adaptive immune recognition [32].

DNA sensing within tumor cells and STING signaling in host cells represent distinct requirements. cGAMP produced by tumor cells can activate STING in nonmalignant cells and support NK cell responses, even when cGAMP production and sensing occur in different cells [15]. Conversely, restoring STING expression in melanoma cells can improve tumor antigenicity and enhance recognition by T cells [23,32]. These findings highlight the importance of assessing individual cellular compartments rather than inferring STING activity in tumor cells from the overall interferon (IFN) signature.

However, cGAS–STING signaling is not uniformly antitumorigenic. Persistent activation driven by chromosomal instability can rewire downstream signaling in cancer cells and promote inflammatory programs associated with immune suppression and metastasis [25]. Thus, the biological consequences of DNA sensing in tumor cells depend not simply on pathway activation, but also on the duration of signaling, the cellular context, and the configuration of downstream signaling networks [7].

### 2.2. RNA Sensing

Cytosolic immunostimulatory RNA can be sensed by the RIG-I-like receptors (RLRs) RIG-I and MDA5. Both receptors signal through the adaptor MAVS, which activates TBK1 and IκB kinase epsilon (IKKε) to promote IRF3 and IRF7 activity and also stimulates NF-κB signaling through the IκB kinase (IKK) complex, thereby inducing type I interferons and other inflammatory genes [33]. RIG-I preferentially recognizes relatively short dsRNA bearing specific end structures, particularly 5′-triphosphate termini, whereas MDA5 preferentially responds to long dsRNA [34,35].

In cancer cells, immunostimulatory RNA can arise from endogenous retroelements and other repetitive sequences, aberrant RNA processing or decay, and mitochondrial RNA [36,37]. Epigenetic dysregulation can further promote transcription from endogenous retroviruses and repetitive elements, leading to the accumulation of dsRNA that is perceived as a viral signal and activates a “viral mimicry” response [38].

Activation of RIG-I/MDA5–MAVS signaling in tumor cells can induce interferon-stimulated genes, chemokines such as CCL5 and CXCL10, and components involved in antigen presentation [9]. These responses can facilitate the recruitment of immune cells and improve immune recognition of tumor cells [9]. Cytoplasmic RNA sensing therefore represents an important route through which intracellular abnormalities in cancer cells can influence antitumor immunity [9,19].

Importantly, cancer cells use multiple mechanisms to prevent endogenous RNA from aberrantly activating cytosolic RNA sensors. RNA editing, RNA decay, and RNA remodeling mediated by helicases can reduce the abundance, structural integrity, or immunogenicity of endogenous dsRNA [19,20,21,36]. ADAR1, XRN1, DHX9, and DDX6 can therefore be viewed as components of a regulatory network that limits the immunogenicity of endogenous RNA and prevents inappropriate activation of innate immune signaling in tumor cells [19,20,21,36]. These mechanisms are discussed in detail in the following section.

### 2.3. Nucleic Acid Sensing in Endosomes

Within endosomes, nucleic acids are primarily recognized by TLR3, TLR7, TLR8, and TLR9. TLR3 senses dsRNA, TLR7 and TLR8 recognize ssRNA and ligands derived from RNA, whereas TLR9 preferentially detects single-stranded DNA containing unmethylated CpG motifs [24,39,40]. TLR3 signals through the TLR adaptor Toll/interleukin-1 receptor domain-containing adapter-inducing interferon-β (TRIF), whereas TLR7/8/9 predominantly use myeloid differentiation primary response 88 (MyD88) to activate distinct transcriptional programs controlled by IRFs and NF-κB [41].

Some TLRs that mediate nucleic acid sensing are expressed and remain functional in malignant cells, although their expression and biological consequences differ considerably according to receptor identity and tumor type [38,42,43]. Their effects are highly context-dependent: tumor cell TLR signaling can induce interferon and inflammatory programs in some settings [38], whereas sustained or aberrantly rewired signaling can promote tumor cell survival, migration, immune evasion, or therapeutic resistance through pathways including NF-κB and Janus kinase (JAK)–signal transducer and activator of transcription (STAT) signaling [42,43].

Compared with the more extensively studied cGAS–STING and RIG-I/MDA5 pathways in tumor cells, the contribution of nucleic acid sensing by TLRs to spontaneous antitumor immunity remains less well defined. Accordingly, the remainder of this review focuses primarily on cytosolic DNA and RNA sensing, for which tumor-associated mechanisms of immune suppression and therapeutic reactivation have been more directly established.

**Figure 1:**
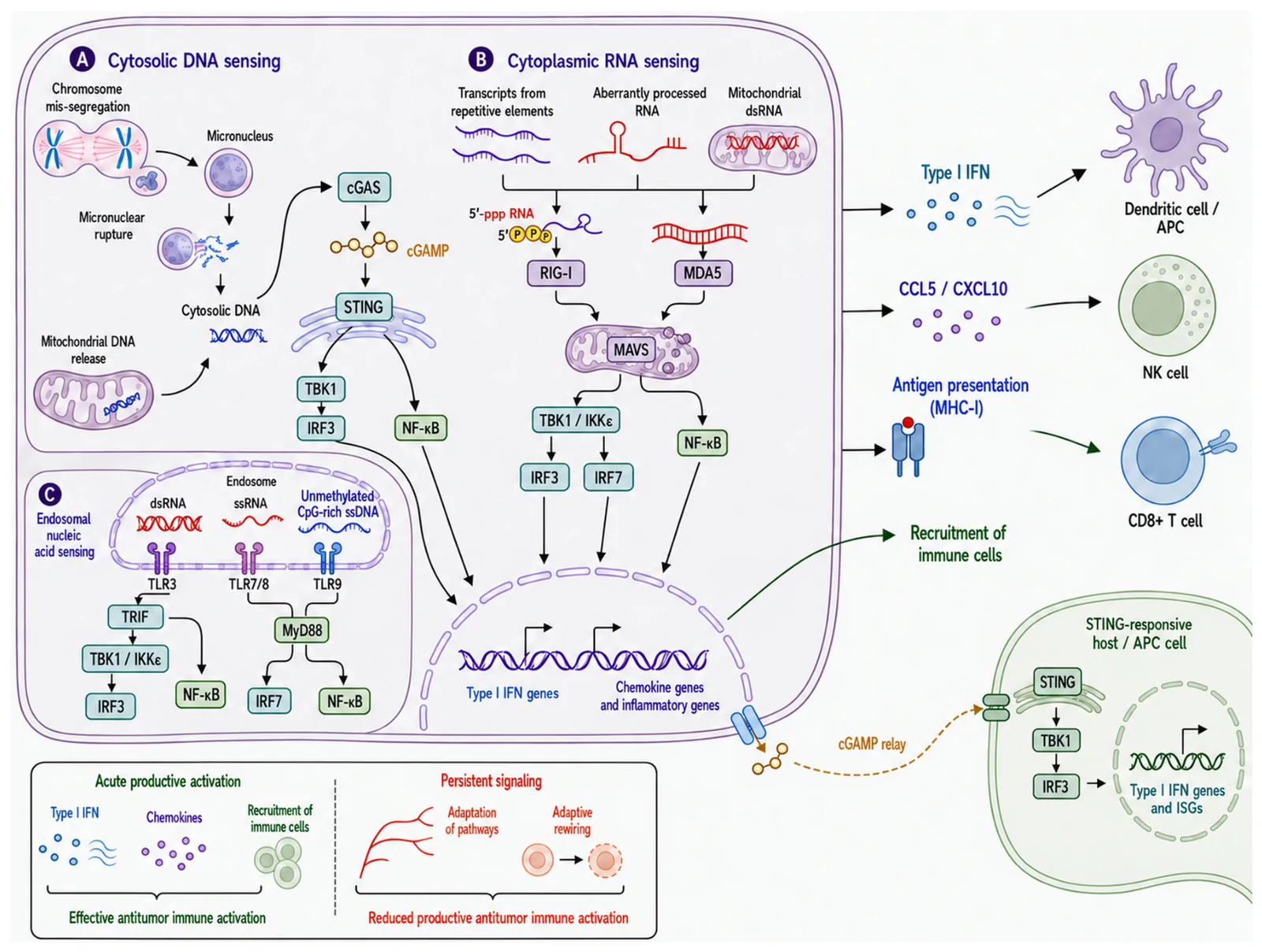
Nucleic acid sensing in tumor cells links intracellular abnormalities to antitumor immunity. Malignant cells can detect aberrant nucleic acids through three major sensing systems. (**A**) Cytosolic DNA sensing: chromosome missegregation, micronuclear rupture, and mitochondrial DNA release generate cytosolic DNA that activates cGAS, leading to cGAMP production, STING activation, and downstream IRF3 and NF-κB signaling. (**B**) Cytoplasmic RNA sensing: transcripts from repetitive elements, aberrantly processed RNA, and mitochondrial dsRNA can activate RIG-I or MDA5, which signal through MAVS. MAVS activates TBK1/IKKε–IRF3/IRF7 signaling and NF-κB through parallel downstream pathways. (**C**) Nucleic acid sensing in endosomes: TLR3 recognizes dsRNA and signals through TRIF to activate TBK1/IKKε–IRF3 and NF-κB, whereas TLR7/8/9 signal predominantly through MyD88 to activate IRF7 and NF-κB. These pathways induce type I interferons, chemokines, and other inflammatory mediators, thereby supporting antigen presentation and the recruitment or activation of dendritic cells, NK cells, and CD8^+^ T cells. cGAMP released from tumor cells can also relay STING signaling to neighboring STING-responsive host cells. The inset illustrates the context dependence of these responses: acute productive activation can support antitumor immunity, whereas persistent signaling may promote adaptive rewiring and reduce productive immune activation. Abbreviations: DNA, deoxyribonucleic acid; RNA, ribonucleic acid; dsRNA, double-stranded RNA; ssRNA, single-stranded RNA; cGAS, cyclic GMP–AMP synthase; cGAMP, cyclic GMP–AMP; STING, stimulator of interferon genes; TBK1, TANK-binding kinase 1; IKKε, IκB kinase epsilon; IRF, interferon regulatory factor; NF-κB, nuclear factor kappa B; RIG-I, retinoic acid-inducible gene I; MDA5, melanoma differentiation-associated protein 5; MAVS, mitochondrial antiviral-signaling protein; TLR, Toll-like receptor; TRIF, Toll/interleukin-1 receptor domain-containing adapter-inducing interferon-β; MyD88, myeloid differentiation primary response 88; CpG, cytosine-phosphate-guanine; IFN, interferon; ISG, interferon-stimulated gene; CCL5, C-C motif chemokine ligand 5; CXCL10, C-X-C motif chemokine ligand 10; MHC-I, major histocompatibility complex class I; NK, natural killer; APC, antigen-presenting cell; CD8, cluster of differentiation 8. The bent arrows above DNA indicate transcription. The 5′-ppp label denotes a triphosphate RNA terminus, and the TLR9 ligand is unmethylated CpG-rich ssDNA.

The major pathways of nucleic acid sensing in tumor cells and their immunological consequences are summarized in Figure 1.

## 3. Molecular Checkpoints That Restrain Innate Immunity in Tumor Cells

Despite extensive genomic and transcriptomic abnormalities, many tumors remain inefficient at activating their own innate sensing pathways. This apparent paradox reflects multiple mechanisms that limit the accumulation or recognition of endogenous immunostimulatory DNA and RNA or restrict the propagation of downstream innate immune signals. Here, the term “molecular checkpoint” is used functionally to describe negative regulators that constrain tumor-associated nucleic-acid sensing; it does not imply clinical equivalence to canonical adaptive immune checkpoints such as PD-1 or CTLA-4. These restraints operate through nucleic-acid clearance, RNA editing and decay, second-messenger degradation, and epigenetic silencing [17,19,22,23].

### 3.1. Nucleic Acid Clearance

One of the most direct strategies for evading innate immune recognition is to eliminate aberrant nucleic acids before they engage cytosolic sensors. In the DNA sensing compartment, three-prime repair exonuclease 1 (TREX1) is a major negative regulator of cGAS–STING signaling. By limiting the accumulation of endogenous cytosolic DNA, TREX1 restrains cGAS activation and downstream type I interferon responses [44,45]. This homeostatic function is essential for preventing inappropriate recognition of self-DNA [44,45], but cancer cells can exploit the same mechanism to suppress tumor-intrinsic STING–IFN signaling [14].

Recent work has established a direct role for TREX1 in tumor immune escape. Tani et al. showed that TREX1 expression in cancer cells is coordinately induced with STING by autocrine interferon (IFN)–STAT1 signaling, forming a negative feedback mechanism that limits amplification of STING–IFN signaling [14]. Genetic inactivation of TREX1 in tumor cells increased the accumulation of immunostimulatory DNA in the cytosol, enhanced interferon signaling through STING, promoted the recruitment of T cells and NK cells, and cooperated with PD-1 blockade to strengthen antitumor immunity across multiple mouse tumor models [14]. These findings position TREX1 as an intracellular brake that helps determine whether endogenous DNA abnormalities are translated into productive antitumor immunity [14,17].

A parallel mechanism operates on the RNA side through the 5′–3′ exoribonuclease XRN1. XRN1 limits the accumulation of endogenous RNAs, including retroelement-derived transcripts capable of forming immunostimulatory dsRNA [36]. Genetic loss of XRN1 increases cytosolic dsRNA and activates antiviral signaling through the MDA5–MAVS pathway and the double-stranded RNA-activated protein kinase R (PKR) [18,36]. In tumor models, XRN1 depletion can induce tumor cell intrinsic interferon responses and potentiate cancer immunotherapy, supporting XRN1 as a candidate therapeutic target [18].

TREX1 and XRN1 therefore illustrate a common principle operating across different classes of nucleic acids: malignant cells can reduce immune visibility by limiting the abundance of endogenous ligands capable of activating innate immune sensors. In this framework, TREX1 primarily buffers cytosolic DNA sensing, whereas XRN1 limits the accumulation of immunostimulatory RNA.

### 3.2. RNA Editing and Processing

Cancer cells do not necessarily need to eliminate endogenous RNA to prevent innate immune recognition. An alternative strategy is to alter its sequence or structural features so that it is less efficiently detected by cytosolic RNA sensors [46]. Adenosine deaminase acting on RNA 1 (ADAR1) is one of the best characterized examples of this mechanism [19,46].

ADAR1 converts adenosine to inosine (A-to-I editing) within endogenous dsRNA, thereby limiting inappropriate recognition of self RNA by MDA5 [46]. Loss of ADAR1 function can activate both MDA5 and PKR through partly distinct mechanisms, with defective A-to-I editing being particularly important for the activation of MDA5 by endogenous dsRNA [19,46,47]. In tumor models, ADAR1 deficiency enhances inflammatory signaling and can restore sensitivity to immune checkpoint blockade, including in tumors with impaired antigen presentation [19].

Recent mechanistic work has refined this model by showing that only a small subset of endogenous cytosolic dsRNAs is strongly immunogenic when left unedited. Sun et al. reported that only a small subset of cytosolic dsRNAs requires editing by ADAR1 to evade recognition by MDA5, and that the immunogenicity of these RNAs is associated with specific sequence features, structural properties, and transcript context [48]. These findings suggest that ADAR1 should not be viewed simply as a global suppressor of all cellular dsRNA, but rather as a selective molecular filter that prevents a limited subset of highly immunostimulatory self-dsRNAs from activating MDA5.

RNA helicases and remodeling factors provide an additional layer of control over endogenous dsRNA. In small cell lung cancer (SCLC), DHX9 suppresses the accumulation of immunostimulatory dsRNA, and its depletion induces cytoplasmic dsRNA accumulation and a tumor-intrinsic interferon response [20]. More recently, DDX6 was shown to interact with ADAR1 and repress editing of adenosines at A:C mismatches in endogenous cytoplasmic dsRNA [21]. This prevents formation of stabilizing I:C pairs and limits dsRNA structural stability and immune recognition [21]. Unlike the editing that protects selected dsRNAs from MDA5 recognition, this substrate-dependent effect shows that A-to-I editing can also stabilize RNA duplexes. Depletion of DDX6 in tumor cells promotes dsRNA accumulation and activates immune responses both within tumor cells and in the surrounding microenvironment [21]. Together, these findings broaden the concept of RNA buffering beyond RNA degradation to include regulation of dsRNA abundance, structure, and editing by RNA helicases.

These RNA regulatory factors differ in their effects on RNA abundance, editing and structure, and should not be treated as interchangeable therapeutic targets.

### 3.3. Restriction of Intercellular cGAMP Signaling

Innate immune signaling can also be restrained after nucleic-acid sensing has occurred. A prominent example is ectonucleotide pyrophosphatase/phosphodiesterase 1 (ENPP1), an extracellular cGAMP hydrolase that limits communication between cGAS-active tumor cells and STING-responsive cells in the tumor microenvironment [49]. Membrane-bound ENPP1 has an extracellular catalytic domain and can be expressed by malignant as well as host cells; it acts on exported cGAMP rather than directly inhibiting intracellular STING. It is included here because it directly regulates the propagation of cGAMP generated by cGAS activity in tumor cells [22,49].

Following cGAS activation, cancer cells can export cGAMP into the extracellular space [49]. Extracellular cGAMP acts as an immunotransmitter that can be taken up by cells in the tumor microenvironment and activate STING signaling in these cells [15,49]. This allows DNA sensing in malignant cells to influence surrounding host cells and broaden the antitumor immune response [15]. ENPP1 hydrolyzes extracellular cGAMP and thereby interrupts this intercellular signaling axis [22,49].

Because ENPP1 hydrolyzes extracellular cGAMP, it has been described as an innate immune checkpoint of the cGAMP–STING axis [22]. Genetic and pharmacological studies show that preserving extracellular cGAMP can enhance STING activation in bystander antigen-presenting cells and strengthen antitumor immunity [22,50]. Thus, ENPP1 illustrates a broader principle of tumor immune evasion: tumors can escape innate immune surveillance not only by suppressing cell-autonomous sensing, but also by limiting the intercellular propagation of innate immune signals.

TREX1 and ENPP1 therefore restrain the broader cGAS–STING axis at distinct stages: TREX1 acts upstream by limiting the availability of cytosolic DNA ligands for cGAS, whereas ENPP1 acts at the intercellular signaling stage by degrading exported cGAMP. This division highlights that innate immune silence can result from defects in both signal generation and signal propagation.

### 3.4. Epigenetic Silencing

An even more fundamental mechanism of immune escape is to reduce or eliminate expression of the sensing machinery itself. In several tumor types, components of the cytosolic DNA sensing machinery, particularly cGAS and STING, are recurrently downregulated or functionally silenced [23,51,52]. Epigenetic repression, including promoter DNA methylation and chromatin-modifying programs, represents an important mechanism underlying this loss of tumor-intrinsic innate immune competence [23,51].

The cGAS–STING pathway provides one of the clearest examples. Promoter hypermethylation can suppress cGAS and STING expression in malignant cells, producing a state in which cytosolic DNA fails to elicit an effective type I interferon response [23,52]. Genome-wide DNA methylation profiling in melanoma has demonstrated coordinated promoter hypermethylation and transcriptional silencing of cGAS and STING [23], while earlier studies in colorectal cancer showed that loss of pathway activity can occur in the absence of major mutations or deletions in either gene [52].

Recent evidence further supports the functional and potentially reversible nature of this epigenetic silencing. In selected cancer cells with epigenetically suppressed cGAS–STING activity, the DNA methyltransferase (DNMT) inhibitor decitabine restored cGAS and STING expression and recovered downstream signaling in response to cytosolic DNA [53]. Decitabine also increased endogenous retroviral transcripts and intracellular dsRNA, thereby activating RIG-I and MDA5 signaling through MAVS [53]. In murine CT26 and 4T1 tumor models, decitabine combined with cisplatin restored cGAS–STING expression in vivo and enhanced antitumor immune activity [53].

Restoration of pathway expression and viral mimicry are separate potential consequences of DNMT inhibition, not a necessary sequence. Their occurrence depends on the epigenetic and signaling state of the tumor, and demethylation cannot replace an irreversibly deleted sensing gene [23,38,53].

Epigenetic regulation can therefore suppress innate immune signaling in tumor cells through two complementary mechanisms: direct silencing of components involved in nucleic acid sensing and reduced transcription of endogenous nucleic acids capable of activating these pathways [23,38,53]. Unlike fixed genetic loss, such epigenetic states are potentially reversible, making them particularly relevant to therapeutic strategies aimed at restoring innate immune competence [23,53].

**Figure 2:**
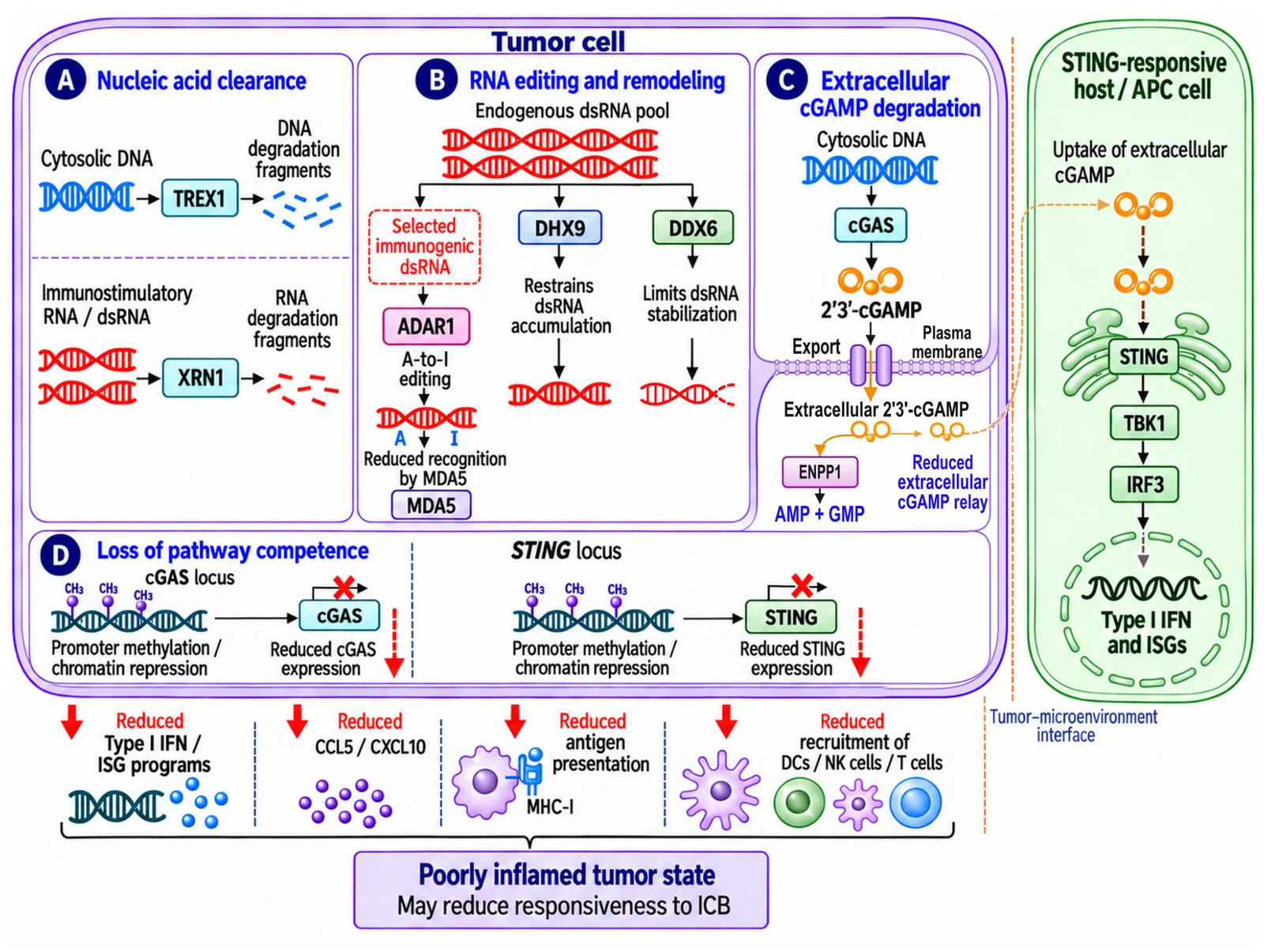
Multilayered molecular checkpoints that suppress nucleic acid sensing in tumor cells. Innate immune signaling can be restrained through several mechanistically distinct processes. (**A**) Nucleic acid clearance: TREX1 degrades cytosolic DNA and thereby limits the availability of DNA for cGAS activation, whereas XRN1 reduces the accumulation of immunostimulatory RNA and dsRNA. (**B**) RNA editing and remodeling: ADAR1 edits a selected subset of immunogenic endogenous dsRNAs, thereby limiting their recognition by MDA5; DHX9 restricts the accumulation of immunostimulatory dsRNA; and DDX6 regulates dsRNA structure, editing state, and immune recognition. (**C**) Extracellular cGAMP degradation: ENPP1 hydrolyzes extracellular 2′3′-cGAMP, ultimately yielding AMP and GMP and reducing the relay of tumor-derived cGAMP to neighboring STING-responsive host or APC cells. (**D**) Loss of pathway competence: promoter methylation and chromatin repression can reduce cGAS and STING expression and impair responses to cytosolic DNA. These mechanisms collectively reduce interferon signaling, chemokine production, antigen presentation, and immune cell recruitment, thereby favoring a poorly inflamed tumor state that may reduce responsiveness to immune checkpoint blockade. The term “molecular checkpoint” is used functionally and does not imply equivalence to canonical adaptive immune checkpoints such as PD-1, PD-L1, or CTLA-4. Abbreviations: DNA, deoxyribonucleic acid; RNA, ribonucleic acid; dsRNA, double-stranded RNA; TREX1, three-prime repair exonuclease 1; XRN1, 5′–3′ exoribonuclease 1; ADAR1, adenosine deaminase acting on RNA 1; A-to-I, adenosine-to-inosine; DHX9, DExH-box helicase 9; DDX6, DEAD-box helicase 6; MDA5, melanoma differentiation-associated protein 5; ENPP1, ectonucleotide pyrophosphatase/phosphodiesterase 1; cGAS, cyclic GMP–AMP synthase; cGAMP, cyclic GMP–AMP; STING, stimulator of interferon genes; AMP, adenosine monophosphate; GMP, guanosine monophosphate; APC, antigen-presenting cell; TBK1, TANK-binding kinase 1; IRF3, interferon regulatory factor 3; IFN, interferon; ISG, interferon-stimulated gene; CCL5, C-C motif chemokine ligand 5; CXCL10, C-X-C motif chemokine ligand 10; MHC-I, major histocompatibility complex class I; DC, dendritic cell; NK, natural killer; ICB, immune checkpoint blockade. PD-1, programmed cell death protein 1; PD-L1, programmed death-ligand 1; CTLA-4, cytotoxic T-lymphocyte-associated protein 4.

These checkpoints constrain ligand availability, RNA recognition, intercellular signal propagation or pathway expression, as summarized in Figure 2.

## 4. From Innate Immune Silencing to Tumor Immune Escape

Suppression of nucleic acid sensing in tumor cells can influence the broader tumor microenvironment. By limiting interferon and chemokine production, reducing the immunogenicity of tumor cells, and impairing communication with surrounding immune cells, it can promote a poorly inflamed tumor state [14,19,31]. This provides an important mechanistic link between the molecular checkpoints described above and the failure of effective adaptive antitumor immunity.

### 4.1. IFN and Chemokine Suppression

Type I interferons are major outputs of DNA and RNA sensing in tumor cells. Activation of the cGAS–STING pathway or signaling through RIG-I, MDA5, and MAVS can induce type I interferons, interferon-stimulated genes, and inflammatory chemokines such as CCL5 and CXCL10 [9,31]. These signals extend the consequences of intracellular nucleic acid sensing beyond the tumor cell by promoting the recruitment and activation of immune populations within the tumor microenvironment [9,31].

Accordingly, suppression of nucleic acid sensing in tumor cells can reduce interferon and chemokine production. Inactivation of TREX1 in tumor cells, for example, enhances STING–IFN signaling and promotes the recruitment of T cells and NK cells [14], whereas relieving restraints on endogenous RNA sensing can similarly activate interferon programs and reshape the surrounding immune microenvironment [20]. Together, these findings support a model in which tumor intrinsic innate immune silencing contributes to poorly inflamed tumors by diminishing signals that promote immune cell recruitment and activation.

The relationship is nevertheless not linear: the strength, duration, and cellular context of interferon signaling shape its biological effects, and chronic signaling can differ substantially from acute immune activation [25,54]. Thus, reduced interferon and chemokine production by tumor cells can facilitate immune escape, whereas persistent interferon signaling may become maladaptive and contribute to immune dysfunction or therapeutic resistance, as discussed below [25,54].

### 4.2. Defective Antigen Presentation and Immune Recruitment

Tumor intrinsic innate immune sensing also influences how effectively malignant cells are recognized by adaptive immunity. Type I interferon signaling downstream of nucleic acid sensing can upregulate components of the antigen processing and presentation machinery, including major histocompatibility complex (MHC) class I and associated antigen processing factors [9,32]. Attenuation of these pathways can therefore reduce both inflammatory communication and the antigen-presenting capacity of tumor cells, thereby limiting their recognition by adaptive immune cells [9,32].

Endogenous nucleic acid sensing can also reinforce antigen presentation programs. Viral mimicry induced by retroelement expression can enhance interferon signaling and increase the expression of genes involved in antigen presentation in tumor cells [9,16]. Conversely, attenuation of these programs can reduce the antigenicity of tumor cells and facilitate immune escape, including under sustained immune pressure from T cells [55].

Suppression of nucleic acid sensing in tumor cells can also disrupt interactions between malignant cells and professional antigen-presenting cells. Type I interferons and chemokines produced by tumor cells can create an inflammatory environment that supports dendritic cell activation, antigen presentation, and the recruitment of T cells [31,56]. When this signaling network is attenuated, tumors may exhibit reduced infiltration and activation of effector lymphocytes, thereby limiting both local immune recognition and the subsequent amplification of adaptive responses [31,56]. In this context, defective antigen presentation and impaired immune recruitment should not be viewed as entirely independent escape mechanisms; rather, they can arise as parallel downstream consequences of a common upstream defect in tumor-intrinsic innate sensing.

The immune effects extend beyond T cells and NK cells. Dendritic cells connect innate activation to antigen presentation, whereas macrophage and neutrophil states can support immune suppression or tissue inflammation as well as tumor control [25,31]. Eosinophils and group 2 innate lymphoid cells provide an additional context-dependent component of the immune ecosystem. As discussed by Wagner et al., type 2 responses can promote tissue repair and tumor growth in some settings but can also support antitumor activity [57]. This broader literature does not establish a direct effect of each nucleic acid checkpoint on these populations; it indicates why recruitment alone cannot define a beneficial immune response.

### 4.3. Resistance to Immune Checkpoint Blockade

The relationship between innate immune silencing and immune checkpoint blockade (ICB) is particularly important because ICB generally acts on pre-existing or inducible antitumor T cell responses rather than generating effective tumor immunity de novo. PD-1/PD-L1 blockade relies particularly on the presence of an existing population of T cells capable of recognizing tumor antigens, whereas CTLA-4 blockade acts on partly distinct T cell populations and can further amplify antitumor responses [58,59]. The efficacy of ICB therefore depends, at least in part, on the presence of lymphocytes capable of recognizing tumor cells, intact antigen presentation, and an inflamed tumor microenvironment that can sustain T cell effector function [55,58].

Tumor-intrinsic nucleic-acid sensing can contribute to each of these prerequisites. Inactivation of TREX1 in cancer cells enhances STING–IFN signaling and cooperates with PD-1 blockade to augment antitumor immunity across multiple tumor models [14]. Likewise, RIG-I signaling in tumor cells has been shown to be required for optimal responses to CTLA-4 blockade in several preclinical cancer models, supporting a functional link between RNA sensing within tumor cells and checkpoint immunotherapy [60]. Together, these findings suggest that nucleic acid sensing can act upstream of the immune context required for effective immune checkpoint blockade.

A useful conceptual distinction can therefore be made between adaptive immune checkpoint inhibition and tumor intrinsic innate immune silencing. In inflamed tumors, PD-1/PD-L1 signaling can restrain existing antitumor T cell responses [58]. By contrast, defective nucleic acid sensing may limit the production of interferons, chemokines, and signals that support antigen presentation, thereby impairing the establishment or maintenance of a T cell-rich immune microenvironment [31,56]. PD-1 blockade alone may therefore be insufficient when the upstream processes required for tumor recognition, antigen presentation, and immune cell recruitment are markedly impaired [31,56].

Clinical studies of ICB-resistant tumors are broadly consistent with this framework, although defects in innate nucleic-acid sensing represent only one potential component of a heterogeneous resistance landscape. In melanoma, acquired resistance to PD-1 blockade has been associated with alterations in interferon signaling and MHC class I antigen presentation [55]. More recent profiling has shown that many lesions resistant to anti-PD-1 therapy are characterized by a poorly inflamed microenvironment with limited infiltration by T cells [61]. Suppression of innate immune activity in tumor cells may therefore contribute to the development of such an immune state, thereby reducing the antitumor immunity required for effective checkpoint blockade.

These findings provide a rationale for testing innate immune reactivation alongside ICB, while recognizing that defective sensing is only one of several causes of resistance.

## 5. Therapeutic Reactivation of Innate Immunity in Tumor Cells

Mechanisms that restrain nucleic acid sensing in tumor cells may simultaneously create opportunities for therapeutic intervention. Restoring this innate immune competence can enhance interferon signaling, inflammatory chemokine production, and immune cell recruitment, thereby shifting poorly inflamed tumors toward a state more permissive to adaptive antitumor immunity [14,31,60]. Therapeutic strategies can be broadly grouped into direct activation of nucleic acid sensing pathways, inhibition of molecular checkpoints that restrain endogenous sensing, and combination approaches that couple innate immune reactivation with established immunotherapies.

The aim is controlled immune reawakening, not indiscriminate amplification of inflammation. The distinction between acute and unresolved inflammation is relevant to treatment design [62], while tumor-specific studies show that persistent cGAS–STING signaling or prolonged IFN exposure can support metastatic or resistance-associated programs [25,54]. These observations do not imply that every chronic response is harmful or that direct STING inhibition is required in all such tumors.

### 5.1. Direct Sensor Activation

The most straightforward approach is direct pharmacological activation of nucleic acid sensing pathways. Among these, STING has been one of the most extensively pursued therapeutic targets. Cyclic dinucleotides and non-nucleotide STING agonists can directly engage STING, activate TBK1–IRF3 signaling, and induce type I interferons and inflammatory mediators [63,64]. In STING-competent tumor cells, this strategy can bypass insufficient endogenous cGAMP production. STING agonists can also act in host antigen-presenting cells, so tumor-cell STING deficiency does not establish host–cell unresponsiveness [15,63].

However, clinical translation of direct STING activation has been challenging. In a phase Ib study of the intratumoral STING agonist MIW815 (ADU-S100) combined with the PD-1 inhibitor spartalizumab, pharmacodynamic analyses confirmed on-target immune activation, yet the objective response rate was only 10.4%, and overall antitumor activity remained limited despite acceptable tolerability [65]. These clinical findings, together with mechanistic studies, point to several potential limitations of direct STING agonism, including constraints imposed by intratumoral delivery and drug retention [65,66], heterogeneous STING competence across tumors [23], incomplete engagement of therapeutically relevant cellular compartments, and the context-dependent consequences of prolonged STING signaling [25]. Collectively, these observations suggest that successful STING-directed therapy may require not simply stronger pathway activation, but more precise control over where, when, and in which cells STING is engaged.

RNA sensing pathways provide an alternative route for therapeutic innate immune activation. Synthetic dsRNA formulations such as polyinosinic:polycytidylic acid [poly(I:C)] can engage endosomal TLR3 and cytosolic MDA5, with RIG-I and other RNA sensors contributing depending on RNA structure and intracellular delivery [67]. A clinically relevant example is BO-112, a nanoplexed synthetic dsRNA administered intratumorally [67]. In the phase II SPOTLIGHT-203 trial in patients with anti-PD-1-resistant advanced melanoma, BO-112 combined with pembrolizumab achieved an objective response rate of 25% in the response evaluable population [68]. Because intratumoral BO-112 can engage both malignant and nonmalignant cellular compartments, however, its clinical activity cannot be attributed specifically to tumor cell intrinsic RNA sensing [67].

Endosomal TLR agonists have also been clinically explored according to a similar principle, particularly TLR9 agonists in combination with immune checkpoint blockade [69]. However, because the antitumor effects of these agents are mediated substantially through immune cell compartments, including plasmacytoid dendritic cells, their activity should not generally be interpreted as tumor cell intrinsic [70]. For the specific goal of restoring cancer cell intrinsic nucleic acid sensing, the cGAS–STING and RIG-I/MDA5 pathways therefore remain more directly relevant therapeutic targets. An important future objective will be to develop delivery systems that concentrate agonists within selected tumor compartments or defined cellular populations, rather than activating nucleic acid sensing indiscriminately throughout the tumor microenvironment [71].

### 5.2. Innate Immune Checkpoint Blockade

An alternative to supplying exogenous agonists is to release endogenous restraints that limit the recognition of tumor-associated nucleic acids or the propagation of their downstream innate immune signals [14,19,22]. This strategy is referred to here as innate immune checkpoint blockade in the functional sense defined in Section 3. Unlike PD-1/PD-L1 blockade, which relieves inhibitory signaling in tumor-reactive lymphocytes, targeting regulators such as TREX1 and ADAR1 aims to release cell-intrinsic brakes on endogenous nucleic acid sensing [14,19], whereas inhibition of ENPP1 preserves extracellular cGAMP and thereby enhances intercellular STING signaling [22].

TREX1 is among the most compelling examples. Because TREX1 degrades cytosolic DNA before it can activate cGAS, TREX1 inhibition allows endogenous tumor-associated DNA to accumulate and engage the cGAS–STING pathway [72]. In 2025, a systematic screen of small molecules identified compound #296 as an inhibitor of TREX1 DNase activity [72]. Pharmacological TREX1 inhibition induced type I IFN signaling in cancer cells, increased intratumoral T cell infiltration, suppressed tumor growth in a type I IFN receptor-dependent manner, and cooperated with immune checkpoint blockade in mouse tumor models [72]. Unlike direct STING agonism, this strategy uses endogenous cytosolic DNA rather than supplying an exogenous STING ligand. Mechanistically, the tumor cell intrinsic effects of TREX1 loss require an intact cGAS–STING pathway, although therapeutic TREX1 inhibition may also engage host immune compartments [72,73].

A separate inhibitor, VB-85680, inhibited human and mouse TREX1 activity in cell lysates and enhanced IFN-related responses in DNA-stimulated THP1 cells [74]. This establishes biochemical and cellular activity, but the reported experiments did not establish tumor control in vivo or clinical efficacy.

ENPP1 provides another attractive checkpoint operating at a distinct level of the cGAS–STING axis. Rather than limiting cGAMP production, ENPP1 hydrolyzes extracellular cGAMP and thereby restricts the propagation of tumor-derived innate immune signals to neighboring STING-responsive cells, including antigen-presenting cells [22,49]. Recent development of selective ENPP1 inhibitors illustrates the therapeutic potential of preserving endogenous extracellular cGAMP. The orally bioavailable ENPP1 inhibitor ISM5939 stabilized extracellular cGAMP, enhanced STING signaling in neighboring antigen-presenting cells, remodeled the tumor immune microenvironment, and synergized with PD-1/PD-L1 blockade across multiple preclinical tumor models [50]. Importantly, this strategy represents an indirect form of STING modulation: rather than directly agonizing STING, ENPP1 inhibition preserves and amplifies cGAMP-dependent signaling generated locally by cGAS-active tumor cells [49,50].

RNA regulatory proteins constitute a parallel class of potential innate immune checkpoints. ADAR1 limits the recognition of selected immunogenic endogenous dsRNAs through A-to-I RNA editing, whereas loss of ADAR1 in tumor cells activates signaling through MDA5 and PKR and can overcome resistance to immune checkpoint blockade [19]. Similarly, XRN1 depletion promotes the accumulation of aberrant immunostimulatory RNA, activates antiviral interferon signaling, and potentiates cancer immunotherapy in experimental models [18]. More recently, DDX6 has emerged as an additional regulator of tumor cell dsRNA sensing. By repressing ADAR1-mediated editing at A:C mismatches, DDX6 limits formation of stabilizing I:C pairs in endogenous dsRNA, whereas DDX6 depletion promotes dsRNA accumulation and activates both tumor cell intrinsic and broader antitumor immune responses [21].

The therapeutic maturity of these targets nevertheless differs substantially. The cited TREX1 compounds have preclinical pharmacological support, and ENPP1 inhibitors have also entered early clinical investigation [50], whereas targets involved in RNA regulation have heterogeneous levels of pharmacological validation [50,72]. Pharmacological targeting of ADAR1 has been reported but remains unsettled [75,76], while XRN1 inhibitor development is still largely at the biochemical and tool compound stage and DDX6 is supported primarily by genetic evidence [21,77]. Importantly, some of these regulators, particularly TREX1 and ADAR1, serve as physiological safeguards against inappropriate recognition of self-nucleic acids [44,46]. Tumor-selective delivery, partial or transient inhibition, and biomarker-guided patient selection may therefore be important for maximizing antitumor activity while minimizing the risk of excessive interferon signaling and systemic inflammatory or autoinflammatory toxicity.

DHX9 illustrates the need to separate pharmacological tractability from immune mechanism. The selective inhibitor ATX968 showed activity in cancer models characterized by mismatch repair deficiency and microsatellite instability, but this finding alone does not establish that its antitumor effects reproduce the immune response driven by dsRNA following DHX9 depletion in SCLC [20,78].

### 5.3. Combination Immunotherapy

The therapeutic value of restoring tumor intrinsic innate sensing may be realized most effectively in combination with adaptive immunotherapies rather than as monotherapy. Innate immune reactivation and immune checkpoint blockade act at complementary stages of the antitumor immune response [14,31]. Innate sensing may provide inflammatory and antigen-presentation signals [14,31], whereas PD-1/PD-L1 blockade can relieve inhibitory signaling in pre-existing tumor-reactive T cells [58]. Thus, innate immune reactivation may establish or restore the immune context required for effective adaptive checkpoint blockade.

This principle is supported by several experimental models. Genetic inactivation or pharmacological inhibition of TREX1 enhances responses to immune checkpoint blockade [14,72], while tumor cell ADAR1 loss can sensitize otherwise resistant tumors to PD-1-directed therapy [19]. ENPP1 inhibition similarly enhances the activity of PD-1/PD-L1 blockade by preserving tumor-derived extracellular cGAMP and reinforcing STING-dependent signaling in antigen-presenting cells [50]. At the clinical level, the activity observed with BO-112 plus pembrolizumab in anti-PD-1 resistant melanoma provides early evidence that therapeutic RNA-sensing activation can induce responses in tumors that have progressed on prior PD-1 directed therapy [68].

Innate immune reactivation may also complement cancer vaccines by enhancing antigen-presenting cell activation and antigen cross-presentation [79], and may improve adoptive cellular therapies by promoting lymphocyte recruitment, effector function, and persistence within tumors [80]. Evidence for these combinations remains predominantly preclinical and is less clinically established than that for combinations with ICB; they should therefore be viewed as emerging therapeutic opportunities rather than validated treatment strategies.

Across combination strategies, a central challenge is achieving the appropriate cellular and temporal pattern of innate immune activation. Chronic STING signaling can rewire downstream responses toward maladaptive inflammatory programs, whereas prolonged interferon signaling can induce compensatory programs associated with immune dysfunction and therapeutic resistance [25,54]. Excessive systemic STING activation may additionally provoke inflammatory toxicity [81]. Targeted delivery platforms may therefore be valuable not only for improving intratumoral drug exposure, but also for limiting systemic immune activation and controlling the cellular compartments in which innate sensing is engaged [71].

**Figure 3:**
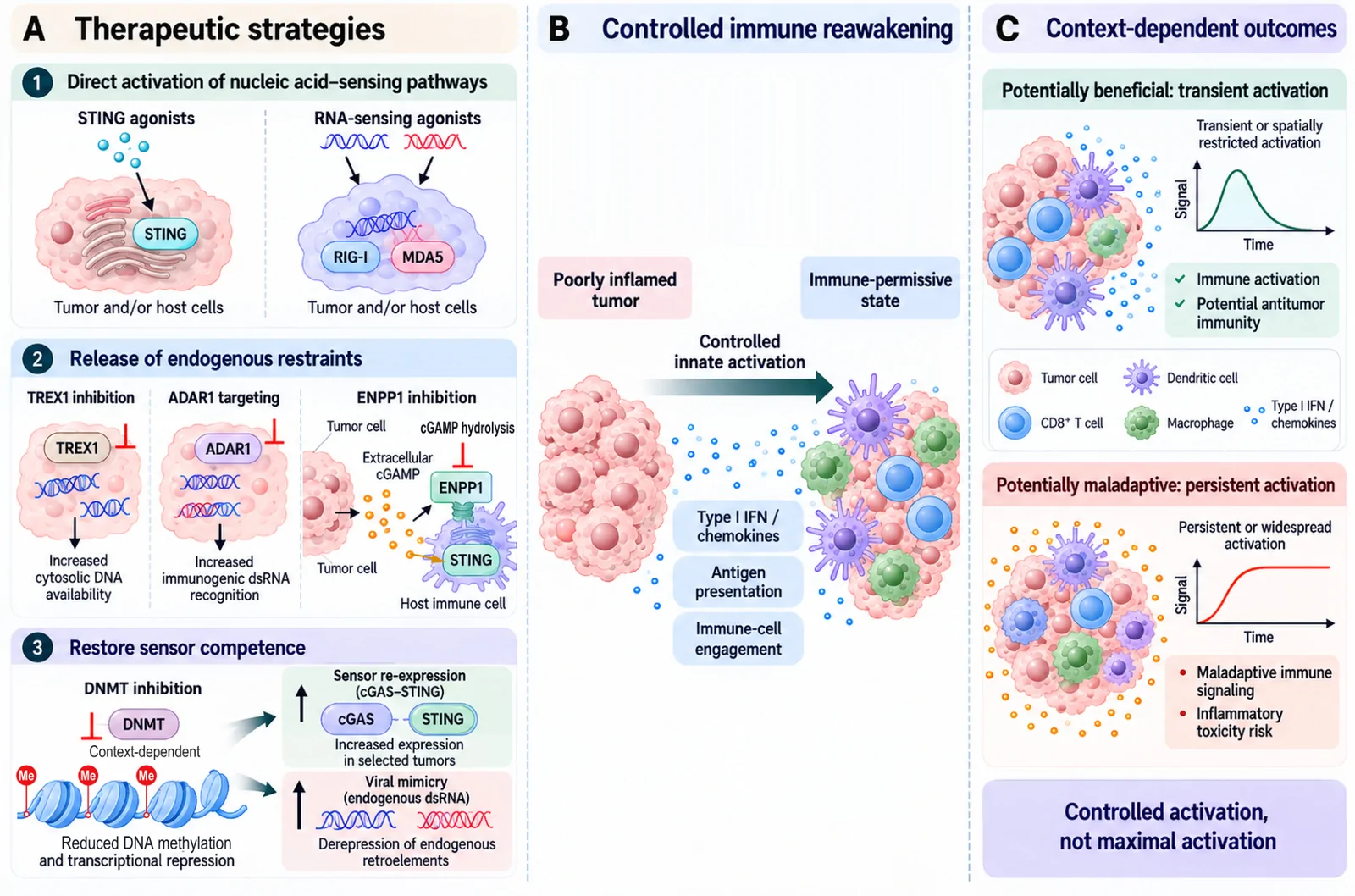
Therapeutic strategies, controlled immune reawakening, and context-dependent outcomes. (**A**) Direct STING or RNA-sensing agonists can act on tumor and/or host cells. TREX1 inhibition increases cytosolic DNA availability, ADAR1 targeting can increase recognition of immunogenic dsRNA, and ENPP1 inhibition reduces extracellular cGAMP hydrolysis, preserving a relay to intracellular STING in competent host cells. ENPP1 is shown in one possible membrane-associated location; its expression is not restricted to host immune cells. DNMT inhibition can relieve DNA methylation-associated repression in selected tumors. Sensor re-expression and endogenous dsRNA-driven viral mimicry are separate potential outcomes and do not imply restoration after irreversible gene loss. Me labels indicate DNA methylation, not histone methylation. (**B**) Controlled innate activation may shift a poorly inflamed tumor toward an immune-permissive state through type I IFN/chemokines, antigen presentation and immune-cell engagement. (**C**) Transient or spatially restricted activation may support antitumor immunity, whereas persistent or widespread activation can produce maladaptive immune signaling or inflammatory toxicity. These outcomes depend on biological context. The two signal–time curves are conceptual illustrations, not experimental data. Abbreviations: STING, stimulator of interferon genes; RIG-I, retinoic acid-inducible gene I; MDA5, melanoma differentiation-associated protein 5; TREX1, three-prime repair exonuclease 1; ADAR1, adenosine deaminase acting on RNA 1; ENPP1, ectonucleotide pyrophosphatase/phosphodiesterase 1; cGAS, cyclic GMP–AMP synthase; cGAMP, cyclic GMP–AMP; DNMT, DNA methyltransferase; DNA, deoxyribonucleic acid; RNA, ribonucleic acid; dsRNA, double-stranded RNA; IFN, interferon; CD8, cluster of differentiation 8; Me, methyl group.

The therapeutic strategies, controlled immune transition and context-dependent outcomes are summarized in Figure 3. Table 1 and Table 2 compare the evidence supporting individual checkpoints and distinguish tumor-cell-intrinsic from host-mediated effects.

## 6. Biomarkers for Precision Targeting of Innate Immunity in Cancer

The heterogeneous functional competence of tumor intrinsic nucleic acid sensing pathways has important implications for therapeutic stratification. Direct sensor agonists and innate immune checkpoint inhibitors are unlikely to benefit all tumors equally, because their activity may depend on an intact sensing and signaling machinery, the abundance and immunogenicity of endogenous nucleic acid ligands, the expression or activity of negative regulators, and the capacity to mount downstream inflammatory responses [14,19,23]. A major translational challenge is therefore to identify tumors in which innate immune sensing is suppressed but remains functionally recoverable [14,23].

The framework below is intended for hypothesis generation. Mechanistic dependency, association with prognosis and prediction of treatment benefit are different levels of evidence, and the integrated framework has not been prospectively validated for selecting patients.

Rather than relying on a single biomarker, the proposed investigational assessment framework integrates sensor competence, suppressor activity, and downstream functional immune output, together with the broader context of cellular identity and spatial organization [14,23,31].

At the sensor level, the expression and functional activity of cGAS, STING, RIG-I, MDA5, MAVS, and related signaling components may indicate whether a tumor retains the molecular machinery required to respond to cytosolic DNA or RNA. This distinction may be particularly important for therapies intended to directly activate tumor cell intrinsic sensing pathways. For example, tumors with epigenetically silenced or otherwise dysfunctional STING signaling may be less capable of mounting a tumor cell intrinsic response to STING-directed stimulation, whereas preserved pathway competence provides a prerequisite for such cell-autonomous activation [23]. However, expression alone does not necessarily indicate functional signaling, and chronic or constitutive pathway activation can have biological consequences distinct from those of an acute, inducible antitumor response [25]. Moreover, large-scale tissue profiling and cell-resolved studies demonstrate substantial heterogeneity in STING expression and activity across tumor types and cellular or spatial compartments, limiting the usefulness of STING expression as a stand-alone biomarker [82,83].

Studies focusing on cancer illustrate both the potential and the limitations of this approach. Qi et al. associated cGAS–STING-related gene expression with prognosis and inferred immune infiltration in hepatocellular carcinoma using public datasets [84]. Chen et al. combined breast cancer tissue staining with transcriptomic analyses and related pathway activation to genomic instability and immune infiltration [85]. These observational findings do not establish prospective prediction of benefit from a sensing-directed drug. In contrast, melanoma studies testing epigenetic restoration and STING stimulation provide functional preclinical evidence that pathway state can alter response [23,32].

At the suppressor level, the expression or activity of negative regulators may provide additional information about the mechanisms underlying innate immune silencing. Elevated activity of TREX1, ENPP1, ADAR1, XRN1, or related regulators may nominate tumors in which innate sensing is actively restrained rather than sensor deficient, although suppressor abundance alone cannot establish downstream pathway competence [14,18,19,22]. Such tumors may be particularly relevant for strategies aimed at releasing innate immune checkpoints. TREX1 provides a useful proof of principle: genetic inactivation or pharmacological inhibition of TREX1 restores tumor cell cGAS–STING–IFN signaling, promotes immune cell infiltration, and enhances the activity of immune checkpoint blockade in preclinical models [14,72]. Importantly, genetic epistasis experiments show that the tumor cell intrinsic interferon response induced by TREX1 loss requires functional cGAS–STING signaling, supporting the need to assess suppressor activity together with downstream pathway competence [72]. Similar principles apply to ADAR1 and other RNA regulatory proteins, for which loss of negative regulation can reactivate endogenous RNA sensing and enhance antitumor immunity, although their value as predictive clinical biomarkers remains unestablished [18,19].

A third, and potentially more informative, layer is the functional output of innate sensing. Type I IFN/ISG signatures, CXCL9/CXCL10/CCL5 expression, antigen presentation programs, and effector immune cell infiltration can provide evidence that nucleic acid sensing is being translated into an immunologically meaningful response [23,31]. Importantly, these downstream features should be interpreted as an integrated functional state rather than as isolated biomarkers. An IFN-associated transcriptional program, for example, may reflect productive acute immune activation or, alternatively, chronic inflammatory adaptation and therapeutic resistance, depending on its duration, cellular source, and signaling context [25,54]. Thus, pathway activity cannot be reliably inferred from a single transcript or protein measurement.

**Figure 4:**
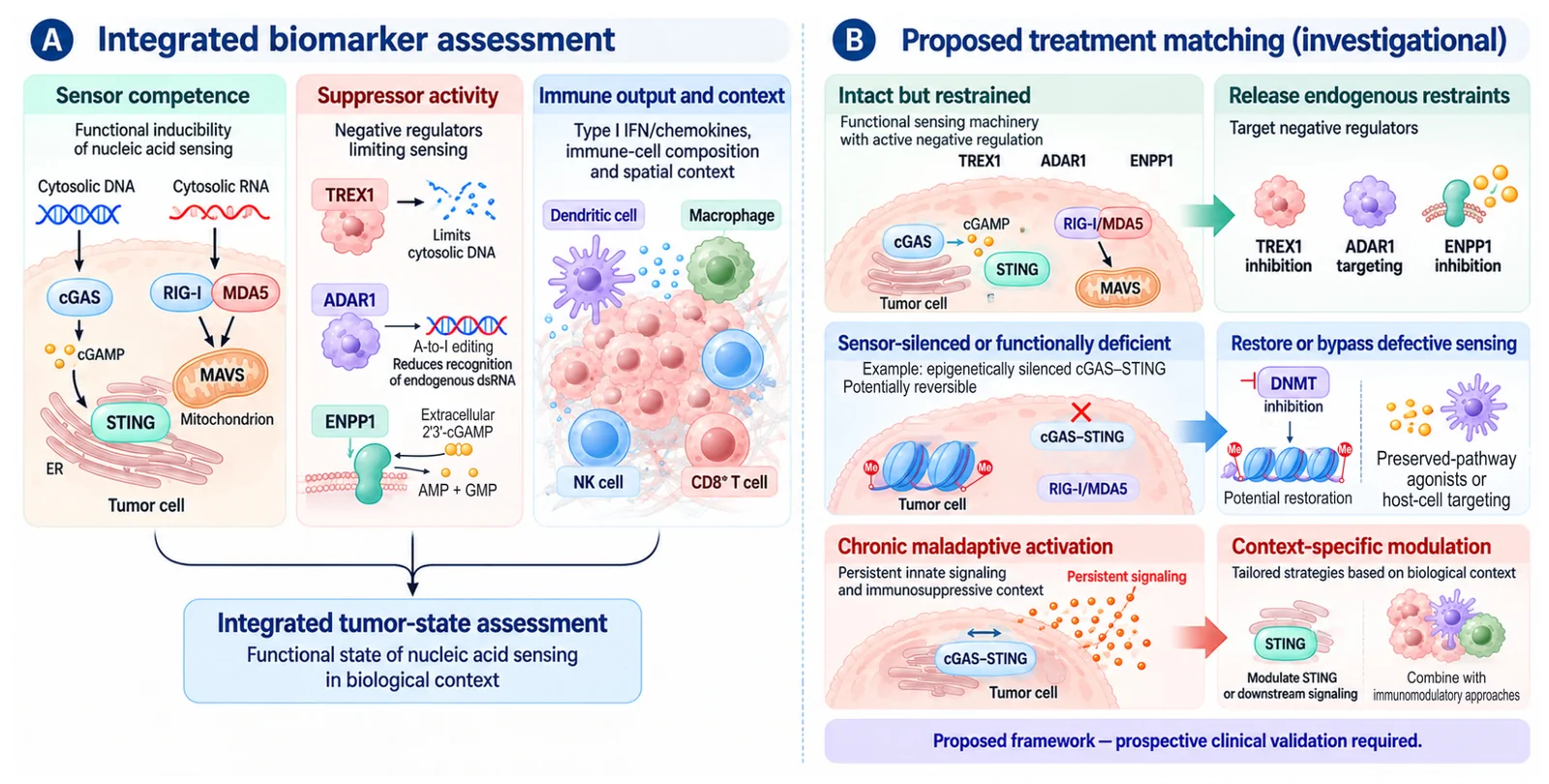
Integrated biomarker assessment and proposed investigational treatment matching. (**A**) Sensor competence, suppressor activity, and immune output and context are integrated to assess the functional state of nucleic acid sensing. Tumor-cell cGAS–STING and RIG-I/MDA5–MAVS pathways are distinct from host–cell sensing. Membrane-associated ENPP1 hydrolyzes extracellular cGAMP to AMP and GMP rather than generating cGAMP or directly regulating STING. (**B**) The proposed directions distinguish intact but restrained sensing, sensor silencing or functional deficiency, and chronic maladaptive activation. cGAS–STING silencing is a representative defect, not evidence that the RNA-sensing pathway is also absent. DNMT inhibition may restore expression after reversible epigenetic repression; Me labels are attached to DNA. Agonists of preserved pathways or host–cell targeting may bypass defects that cannot be restored. Chronic maladaptive signaling calls for context-specific modulation, not obligatory direct inhibition of STING. Arrows indicate investigational hypotheses, not validated treatment-selection rules. The integrated framework requires prospective clinical validation. Abbreviations: cGAS, cyclic GMP–AMP synthase; cGAMP, cyclic GMP–AMP; STING, stimulator of interferon genes; RIG-I, retinoic acid-inducible gene I; MDA5, melanoma differentiation-associated protein 5; MAVS, mitochondrial antiviral-signaling protein; TREX1, three-prime repair exonuclease 1; ADAR1, adenosine deaminase acting on RNA 1; A-to-I, adenosine-to-inosine; ENPP1, ectonucleotide pyrophosphatase/phosphodiesterase 1; DNMT, DNA methyltransferase; DNA, deoxyribonucleic acid; RNA, ribonucleic acid; dsRNA, double-stranded RNA; AMP, adenosine monophosphate; GMP, guanosine monophosphate; ER, endoplasmic reticulum; IFN, interferon; NK, natural killer; CD8, cluster of differentiation 8; Me, methyl group.

Integrating these complementary layers may provide a more informative basis for therapeutic stratification. For example, elevated TREX1 may indicate a therapeutically suppressible DNA sensing state only when downstream cGAS–STING signaling remains functional; similarly, the biological consequences of ADAR1 targeting depend on intact downstream dsRNA sensing pathways, particularly signaling through MDA5 and PKR. Such stratification could distinguish tumors with preserved sensor competence but active suppression from those with impaired sensing that may require restoration of pathway activity or alternative immune strategies. Figure 4 presents integrated biomarker assessment (Panel A) and proposed investigational treatment matching (Panel B). Its arrows indicate hypotheses for evaluation, not validated clinical decision rules.

Reversible epigenetic silencing may justify testing restoration of expression, whereas irreversible loss or failure of tumor-cell STING may require agonists of preserved pathways or strategies directed at competent host cells. A tumor-cell defect in one pathway should not be interpreted as concurrent loss of DNA and RNA sensing.

## 7. Challenges and Future Perspectives

Despite growing evidence that nucleic acid sensing in tumor cells can influence antitumor immunity and therapeutic responses, major challenges remain before these pathways can be translated into precision approaches for cancer immunotherapy. These include defining the cellular sources and spatial organization of innate immune signaling [83], understanding how the effects of pathway activation depend on cellular context and duration [25], establishing a safe therapeutic window for releasing innate immune checkpoints [44,72], and developing clinically validated predictive biomarkers.

### 7.1. Cellular and Spatial Resolution

A major challenge is determining the cellular origin of nucleic acid sensing signatures within tumors. Bulk transcriptomic or proteomic profiling cannot reliably distinguish STING, RIG-I, interferon-response genes, or inflammatory chemokines expressed by malignant cells from the same signals arising from dendritic cells, macrophages, endothelial cells, or lymphocytes [83]. This distinction is critical because activation or expression of the same innate immune pathway in different cellular compartments can have distinct, and in some cases opposing, biological consequences [60,86].

Single-cell RNA sequencing can help resolve whether an innate-sensing program originates from malignant or nonmalignant cellular compartments, whereas spatial transcriptomics and multiplex imaging can reveal how these programs are organized in relation to dendritic cells, NK cells, and cytotoxic T cells [83,87]. Recent single-cell spatial analyses of treated tumors have further shown that regions with distinct immune states can coexist within the same lesion, even after immunotherapy [88]. Future biomarker studies should therefore integrate pathway activity with cell identity and tissue organization rather than relying on bulk expression alone.

### 7.2. Context and Temporal Dynamics

A second challenge is that innate immune activation is not uniformly beneficial. The magnitude, duration, and cellular compartment of signaling can fundamentally alter its biological consequences. Acute activation of cGAS–STING or RIG-I/MDA5–MAVS pathways in tumor cells can induce type I interferon responses, promote inflammatory chemokine production, and facilitate the recruitment of immune cells [9,31]. In contrast, persistent STING and downstream interferon signaling can undergo adaptive rewiring, producing biological outcomes distinct from those of acute antitumor activation [25,54].

This principle is particularly evident in chromosomally unstable tumors. Chromosomal instability can generate cytosolic DNA and chronically engage tumor cell cGAS–STING signaling [30]. Persistent cGAS–STING activation in this setting can drive downstream signaling rewiring, including type I interferon tachyphylaxis, and reshape the tumor microenvironment toward immune suppressive and pro-metastatic states [25]. These observations argue against a simple model in which greater STING or interferon activity invariably predicts stronger antitumor immunity [25].

Therapeutic strategies should therefore aim for appropriately localized and temporally controlled innate immune activation rather than maximal pathway stimulation [25,71]. Defining the differences between acute and chronic signaling, as well as between tumor cell and immune cell activation, will be essential for identifying the conditions under which innate sensing promotes effective and durable antitumor immunity [25,83].

### 7.3. Therapeutic Window and Safety

Several candidate innate immune checkpoints are attractive therapeutic targets because they prevent inappropriate recognition of endogenous nucleic acids. However, this physiological role also creates an important safety concern. TREX1 and ADAR1, in particular, are key safeguards of self nucleic acid tolerance: TREX1 limits aberrant sensing of endogenous DNA, whereas ADAR1 prevents endogenous dsRNA from activating MDA5-dependent innate immune signaling [44,46]. Genetic disruption of these pathways can produce sustained type I interferon activation and autoinflammatory or autoimmune phenotypes in mice and humans [44,47,89,90]. Thus, therapeutic inhibition carries a potential risk of systemic inflammatory toxicity, although the consequences of partial, transient, or tumor-directed pharmacological inhibition may differ substantially from those of germline or complete genetic loss.

Encouragingly, recent preclinical studies suggest that this safety challenge may be manageable. In 2025, inducible whole-body TREX1 inactivation in adult mice was used to model therapeutic systemic TREX1 inhibition. Sustained TREX1 loss produced antitumor activity across multiple tumor models without severe immune toxicity under the conditions tested, including when combined with immune checkpoint blockade [72]. Nevertheless, these preclinical findings do not establish the safety of systemic TREX1 inhibition in humans.

The safety challenge may be particularly complex for RNA regulatory proteins. ADAR1, for example, restrains inappropriate recognition of endogenous dsRNA through distinct mechanisms: loss of its RNA editing activity activates MDA5, whereas disruption of the cytoplasmic ADAR1p150 isoform or its dsRNA-binding function can permit PKR activation and severe autoinflammatory signaling [91]. These findings underscore the need to preserve essential physiological ADAR1 functions while exploiting its tumor-promoting activities. Recent preclinical studies suggest that selective targeting of specific ADAR1 isoforms or domains, particularly the ADAR1p150–Zα axis, may offer a strategy to achieve a more favorable therapeutic index [92]. More broadly, future therapeutic development may require tumor-selective delivery, partial or transient inhibition, or carefully controlled treatment schedules that induce sufficient antitumor innate activation while preserving systemic immune homeostasis.

### 7.4. Clinical Validation and Precision Treatment Matching

A final challenge is translating mechanistic biomarkers into clinically validated predictors of therapeutic response. Although STING pathway status and regulators such as TREX1, ENPP1, and ADAR1 are increasingly supported by mechanistic and translational evidence [14,19,22,23], none has yet been prospectively validated as a stand-alone predictive biomarker for selecting patients for innate immune-directed therapy. Early clinical studies of STING agonists have incorporated exploratory pharmacodynamic biomarkers, but evidence of pathway activation has not yet yielded a validated patient selection marker [65].

The central challenge is not simply to measure a single molecule, but to determine whether a tumor retains a functionally recoverable innate sensing state. Predictive biomarkers will therefore need to distinguish pathway competence from active pathway suppression and to establish whether molecular reactivation produces the intended downstream immune response [23,31,72].

Prospective studies should incorporate sampling before and during treatment, functional assays of pathway activity, profiling at the cellular and spatial levels, and longitudinal clinical outcomes. Such designs will be necessary to determine which tumor states predict benefit from sensor agonists, inhibitors of innate immune checkpoints, or combination immunotherapy.

Ultimately, precision innate immune oncology will require therapies to be matched to the mechanisms underlying innate immune silencing. Tumors with intact but actively restrained sensing may be candidates for inhibition of negative regulators, whereas sensor-deficient or silenced tumors may require restoration of pathway competence or alternative immune strategies. By contrast, tumors with chronic maladaptive innate activation may require context-specific modulation rather than further pathway amplification. These proposed states require prospective testing before they can guide patient treatment.

## 8. Conclusions

Tumor intrinsic nucleic acid sensing links cancer cell abnormalities to immune recognition, but tumors can restrain these responses through nucleic acid clearance, RNA editing and remodeling, extracellular cGAMP degradation, and epigenetic silencing.

These checkpoints differ in their tumor context, experimental support and therapeutic maturity, and genetic evidence should not be equated with established pharmacological or clinical efficacy.

Therapeutic reactivation should account for both tumor and host–cell competence and seek appropriately localized, temporally controlled signaling rather than maximal activation.

Integrating sensor competence, suppressor activity and functional immune output offers an investigational framework for treatment matching that requires prospective validation before clinical use.

## Figures and Tables

**Table 1:** Checkpoint mechanisms, evidence and translational limitations.

Checkpoint and Sensing Pathway	Tumor Context and Experimental Evidence	Intervention and Stage of Cited Evidence	Major Safety Concern or Limitation
TREX1 cGAS–STING	Cancer-cell deletion supports causality in lung cancer and other mouse models; compound #296 was tested in colon carcinoma and melanoma models [14,72].	Compound #296: preclinical antitumor activity. VB-85680: biochemical and cellular assays, without in vivo tumor efficacy in that report [74].	Self-DNA-driven inflammation; mouse tolerability does not establish human safety [44,72].
XRN1 RNA sensing	Tumor-cell depletion activates endogenous RNA sensing and enhances immunotherapy in mouse models; retroelement decay is a mechanistic dependency [18,36].	Adenosine-3′,5′-bisphosphate: biochemical enzyme inhibitor; genetic tumor evidence exceeds pharmacological validation [77].	Disruption of RNA homeostasis and systemic antiviral signaling; therapeutic window not established.
ADAR1 MDA5/PKR	Tumor-cell loss restores checkpoint sensitivity in mouse melanoma models; effects depend on substrate and downstream sensor competence [19,48].	ZYS-1 has conflicting target-validation reports; selective pharmacological immune targeting remains unsettled [75,76].	Loss of self-RNA tolerance; editing and dsRNA-binding effects are not equivalent [46,47].
DHX9 Endogenous RNA	Genetic depletion triggers dsRNA/IFN and replication stress in SCLC [20]. ATX968 activity in mismatch repair-deficient tumors does not by itself prove immune reactivation [78].	ATX968: selective preclinical tool inhibitor; the cited drug and immune-mechanism studies address different contexts [20,78].	Effects on replication and RNA/DNA homeostasis; human immune therapeutic window unestablished.
DDX6 Endogenous dsRNA	Tumor-cell depletion supports immune activity; suppression of A:C editing limits stabilizing I:C pairs [21].	The cited study provides genetic/mechanistic evidence, rather than a validated therapeutic inhibitor [21].	Broad RNA regulation; drug selectivity and systemic safety remain unresolved.
ENPP1 Extracellular cGAMP relay	Breast cancer genetic studies and multi-model inhibitor studies support extracellular cGAMP preservation and host–cell STING signaling [22,50].	ISM5939: published preclinical pharmacology. ENPP1 inhibitors have entered phase I investigation; clinical benefit is not established [50].	Extracellular nucleotide homeostasis and systemic inflammation; selectivity and exposure require evaluation.
DNA methylation Sensor expression/viral mimicry	Melanoma pathway restoration and CT26/4T1 combination models support context-dependent reversibility [23,53].	Decitabine: clinically used in hematologic malignancy, but restoration-based matching in the solid-tumor contexts cited here is preclinical [53].	Broad epigenetic effects and hematologic toxicity; cannot reverse gene deletion.

Abbreviations: cGAS, cyclic GMP–AMP synthase; STING, stimulator of interferon genes; TREX1, three-prime repair exonuclease 1; XRN1, 5′–3′ exoribonuclease 1; ADAR1, adenosine deaminase acting on RNA 1; DHX9, DExH-box helicase 9; DDX6, DEAD-box helicase 6; ENPP1, ectonucleotide pyrophosphatase/phosphodiesterase 1; cGAMP, cyclic GMP–AMP; MDA5, melanoma differentiation-associated protein 5; PKR, protein kinase R; SCLC, small cell lung cancer; IFN, interferon; DNA, deoxyribonucleic acid; RNA, ribonucleic acid; dsRNA, double-stranded RNA; A, adenosine; I, inosine; C, cytidine.

**Table 2:** Tumor-cell-intrinsic and host-mediated contributions to sensing-directed strategies.

Strategy or Mechanism	Tumor-Cell-Intrinsic Evidence	Host–Cell Contribution	Interpretive Limit
TREX1/RNA regulatory targeting	Tumor-cell genetic perturbations directly test sensing dependencies [14,18,19,20,21].	Immune recruitment and tumor control also require interactions with host cells.	Genetic dependency does not establish drug selectivity or clinical efficacy.
STING agonists	Competent tumor-cell STING can alter antigenicity and treatment response [32].	Host STING can sense tumor-derived cGAMP; agonists also engage nonmalignant compartments [15,63].	Tumor STING loss does not demonstrate host STING loss.
ENPP1 inhibition	Tumor cGAS can provide exported cGAMP; ENPP1 is not an intracellular STING inhibitor [22,49].	Preserved cGAMP can activate STING in host antigen-presenting cells [50].	Activity depends on ligand production, transport and recipient competence, not tumor STING alone.
Intratumoral dsRNA/BO-112	Synthetic dsRNA can engage tumor-cell RNA sensors and inflammatory responses [67].	Immune and stromal compartments may also contribute to intratumoral activity [67].	The phase II combination study does not isolate the tumor-cell-intrinsic component [68].
TLR9 agonists	Tumor-cell receptor expression is heterogeneous and is not sufficient to assign clinical activity.	Plasmacytoid dendritic cells and other immune cells are major effectors [70].	Clinical trials test whole-tumor effects; a phase III combination did not establish added benefit [69].

Abbreviations: TREX1, three-prime repair exonuclease 1; STING, stimulator of interferon genes; ENPP1, ectonucleotide pyrophosphatase/phosphodiesterase 1; cGAS, cyclic GMP–AMP synthase; cGAMP, cyclic GMP–AMP; dsRNA, double-stranded RNA; TLR9, Toll-like receptor 9.

## Data Availability

No new data were created or analyzed in this study. Data sharing is not applicable to this article.

## Abbreviations

ADAR1	Adenosine deaminase acting on RNA 1
ADAR1p150	150-kDa cytoplasmic isoform of ADAR1
AMP	Adenosine monophosphate
APC	Antigen-presenting cell
A-to-I	Adenosine-to-inosine
CCL5	C-C motif chemokine ligand 5
CD8	Cluster of differentiation 8
cGAMP	Cyclic GMP–AMP
2′3′-cGAMP	2′3′-cyclic GMP–AMP
cGAS	Cyclic GMP–AMP synthase
CpG	Cytosine-phosphate-guanine dinucleotide
CTLA-4	Cytotoxic T-lymphocyte-associated protein 4
CXCL9	C-X-C motif chemokine ligand 9
CXCL10	C-X-C motif chemokine ligand 10
DC	Dendritic cell
DDX6	DEAD-box helicase 6
DHX9	DExH-box helicase 9
DNA	Deoxyribonucleic acid
DNase	Deoxyribonuclease
DNMT	DNA methyltransferase
dsDNA	Double-stranded DNA
dsRNA	Double-stranded RNA
ENPP1	Ectonucleotide pyrophosphatase/phosphodiesterase 1
GMP	Guanosine monophosphate
ICB	Immune checkpoint blockade
IFN	Interferon
IKK	IκB kinase
IKKε	IκB kinase epsilon
IRF	Interferon regulatory factor
IRF3	Interferon regulatory factor 3
IRF7	Interferon regulatory factor 7
ISG	Interferon-stimulated gene
JAK	Janus kinase
MAVS	Mitochondrial antiviral-signaling protein
MDA5	Melanoma differentiation-associated protein 5
MHC	Major histocompatibility complex
MHC-I	Major histocompatibility complex class I
MyD88	Myeloid differentiation primary response 88
NF-κB	Nuclear factor kappa B
NK	Natural killer
PD-1	Programmed cell death protein 1
PD-L1	Programmed death-ligand 1
PKR	Protein kinase R (double-stranded RNA-activated protein kinase)
RIG-I	Retinoic acid-inducible gene I
RLR	RIG-I-like receptor
RNA	Ribonucleic acid
SCLC	Small cell lung cancer
ssRNA	Single-stranded RNA
STAT	Signal transducer and activator of transcription
STAT1	Signal transducer and activator of transcription 1
STING	Stimulator of interferon genes
TBK1	TANK-binding kinase 1
TLR	Toll-like receptor
TLR3	Toll-like receptor 3
TLR7	Toll-like receptor 7
TLR8	Toll-like receptor 8
TLR9	Toll-like receptor 9
TREX1	Three-prime repair exonuclease 1
TRIF	Toll/interleukin-1 receptor domain-containing adapter-inducing interferon-β
XRN1	5′–3′ exoribonuclease 1
Zα	Z-DNA/RNA-binding alpha domain of ADAR1
